# ROS from Physical Plasmas: Redox Chemistry for Biomedical Therapy

**DOI:** 10.1155/2019/9062098

**Published:** 2019-10-08

**Authors:** Angela Privat-Maldonado, Anke Schmidt, Abraham Lin, Klaus-Dieter Weltmann, Kristian Wende, Annemie Bogaerts, Sander Bekeschus

**Affiliations:** ^1^PLASMANT Research Group, University of Antwerp, Antwerp, Belgium; ^2^ZIK plasmatis, Leibniz Institute for Plasma Science and Technology (INP Greifswald), Greifswald, Germany; ^3^Center for Oncological Research, University of Antwerp, Antwerp, Belgium

## Abstract

Physical plasmas generate unique mixes of reactive oxygen and nitrogen species (RONS or ROS). Only a bit more than a decade ago, these plasmas, operating at body temperature, started to be considered for medical therapy with considerably little mechanistic redox chemistry or biomedical research existing on that topic at that time. Today, a vast body of evidence is available on physical plasma-derived ROS, from their spatiotemporal resolution in the plasma gas phase to sophisticated chemical and biochemical analysis of these species once dissolved in liquids. Data from *in silico* analysis dissected potential reaction pathways of plasma-derived reactive species with biological membranes, and *in vitro* and *in vivo* experiments in cell and animal disease models identified molecular mechanisms and potential therapeutic benefits of physical plasmas. In 2013, the first medical plasma systems entered the European market as class IIa devices and have proven to be a valuable resource in dermatology, especially for supporting the healing of chronic wounds. The first results in cancer patients treated with plasma are promising, too. Due to the many potentials of this blooming new field ahead, there is a need to highlight the main concepts distilled from plasma research in chemistry and biology that serve as a mechanistic link between plasma physics (how and which plasma-derived ROS are produced) and therapy (what is the medical benefit). This inevitably puts cellular membranes in focus, as these are the natural interphase between ROS produced by plasmas and translation of their chemical reactivity into distinct biological responses.

## 1. Introduction to Cold Physical Plasma

The advancement in medicine could not have been possible without the introduction of innovative technologies from the field of physics to improve the diagnosis and treatment of patients. From radiation therapy to magnetic resonance imaging, these technologies have revolutionised medicine, which allow clinicians to use advanced imaging methods and sophisticated therapies to treat patients. In the last decades, another technology from the physics disciplines has gained visibility: physical plasma. Commonly referred to as the fourth state of matter [1], plasma brings multiple opportunities for patient care that range from cosmetic procedures to clinically relevant pathologies (being the focus of this review) such as wound healing and cancer treatment.

**Figure 1:**
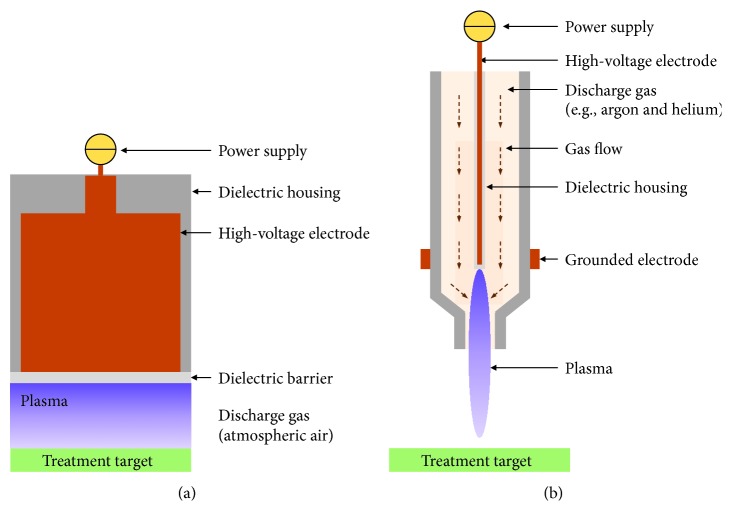
Schematic of two categories of commonly used plasma devices for medical application: dielectric barrier discharges and plasma jets. In dielectric barrier discharges, plasma is generated in atmospheric air directly onto the biological target (a), while in plasma jets, plasma is generated inside the device and delivered to the target via a flow of gas (b).

Cold physical plasma, from here on referred to as plasma, is generated by supplying energy to a gas to induce partial ionization. For medical purposes, there are two main principles, despite some sources not falling into the following categories: (i) dielectric barrier discharges (DBD) that are directly operated in ambient air and (ii) plasma jets that ionize a stream of noble or inert gas that subsequently interacts with oxygen and nitrogen of ambient air. DBDs generate plasma in atmospheric air directly onto the treatment target (Figure 1(a)). A high-voltage pulse is applied to an electrode covered with an insulating barrier and brought near the target, which acts as the second electrode. The barrier reduces the current that is passed to the tissue, making the plasma generated in the gap between the electrodes, thermally and electrically safe [2]. The electrodes used for DBD systems could be fabricated for different sizes, making them ideal for large surface treatments. While several plasma jet configurations are available, they operate on the principle that the bulk of the plasma is generated within the plasma device (Figure 1(b)), which is filled with a constant flow of discharge gas or gas mixture (e.g., argon, helium, and nitrogen) [3]. The generated plasma protrudes from the aperture of the device and is brought in contact with the biological target for treatment. The cross-section of this “plasma plume” is on the order of micrometers, which allows for high-precision treatment.

Common to both principles is the presence of free electrons and ions, free radicals, and neutral molecules in constant interaction [4]. Plasmas operated at ambient pressure and body temperature are of particular interest in biomedicine. The major biologically active component of plasma is the variety of reactive oxygen and nitrogen species formed upon reaction with molecules (oxygen, nitrogen, and water) present in the ambient air [5–7]. Plasma-derived reactive species can be divided into reactive oxygen species, such as ozone (O_3_), superoxide (O_2_^•-^), singlet delta oxygen (^1^O_2_), atomic oxygen (O), hydroxyl radical (^•^OH), and hydrogen peroxide (H_2_O_2_) on the one hand, and reactive nitrogen species, such as nitrogen dioxide radical (^•^NO_2_), peroxynitrite (ONOO^−^), and nitric oxide (^•^NO) on the other [8–10]. Since all the biologically relevant RNS also contain oxygen, we will use the term ROS in this review to refer to both ROS and RNS.

ROS have been acknowledged as the main active agents responsible for the biological effects of direct and indirect plasma treatments (the latter refers to treating a liquid with plasma that is subsequently transferred to cells or tissues) [6, 11, 12]. Other physical components produced by plasma (UV photons and electromagnetic fields) seem to have a negligible cellular impact on their own [13–15] at the intensities generated with plasmas. However, their ability to exert biological effects in cells during direct plasma treatments should not be overlooked. There is evidence that exposure of cells to low electromagnetic field frequencies can induce transient changes in protein [16] and mRNA levels [17], decrease cell proliferation [18], and increase free radical levels [19]. Further studies on the effect of the physical components of plasma other than ROS are needed to elucidate their specific roles.

An advantage of plasma technology is the ability to exert different biological responses based firstly on the type of ROS delivered and secondly by their quantity. ROS have a crucial role in physiological functions, and they can induce different effects on cells depending on their nature, levels, and localization [20]. In medicine, the potential of ROS is being exploited in therapies in, e.g., dermatology, oncology, and dentistry. Direct plasma treatments benefit from the presence of highly active, short-lived ROS produced during ionization, which present a unique chemical opportunity to modulate the responses in target cells. The success of these therapies will depend on the ability of plasma to induce the desired effect in the target tissue, for which it is necessary to understand the underlying mechanisms of action.

To set the stage for a discussion of the future of plasma in the medical field, we outline the theories proposed to account for the effects of plasma-generated ROS and the corresponding signalling pathways at the cellular level. To understand the mechanistic link between plasma and its therapeutic effect, we will focus on the interactions occurring at the membrane microenvironment and the translation of such events into biological responses. The ultimate goal in plasma medicine should be to identify specific types and quantities of plasma-derived ROS (based on either different plasma sources or different operational settings for one plasma source) for the treatment of a specific pathological condition.

## 2. Plasma-Derived ROS in Medical Therapy

The spatiotemporal distribution of the ROS output of some plasma sources like the kINPen is exceptionally well characterized [21]. Naturally, more investigations are needed for this and other types of plasma sources, but there is a certain degree of consent on what ROS plasma sources typically generate and how this can be tuned by changing the feed and ambient gas composition. The medical effects of plasma treatment in patients are promising in dermatology and cancer, as briefly outlined below. For a comprehensive overview of other areas of medical application, the reader is referred to a recent text book covering all aspects of plasma medicine [22].

### 2.1. Dermatology and Skin-Based Infections

Nonhealing wounds are a devastating problem for patients and healthcare systems alike [23]. The increasing incidence of diabetes mellitus as a major ailment for diabetic foot ulcers, as well as the increase in human life expectancy, is likely to magnify this issue [24]. More than a decade ago, it was hypothesized that wound healing is subject to redox control [25–27]. As plasmas emit ROS, it was natural to test their potential effect on nonhealing wounds. Several clinical observations and studies found not only an antimicrobial activity but also a wound healing promoting activity of plasma treatment in acute as well as chronic wounds [28–35] and driveline infections [36]. Using hyperspectral imaging, an increase in wound oxygenation and blood flow was found immediately after plasma treatment [37]. Yet, the efficacy of plasma therapy varies between patients. In general, the evidence level of the majority of clinically relevant wound therapies is low [38]. Part of this problem is a lack of standardization of wound location, size, microbial colonization, and etiology as well as varying treatment procedures prior to hospitalization. Hence, a limited number of randomized clinical trials (RCTs) as well as clinical trials without randomization is reported. Due to the nature of cold physical plasma, blinding the investigators (or patients) is hardly achievable. For the medical product *PlasmaDerm* (NCT01415622), improved wound healing was reported [39]. For the medical product *MicroPlaSter*, three nonregistered RCTs showed a reduction in bacterial load and a modest improvement in wound healing [40–43], while no improvement in patients with pruritus was observed [44]. For the same device, one trial on biofilm removal in diabetic ulcers is ongoing (ISRCTN17491903). For pressure ulcers, another unregistered trial reported a reduction in microbial burden and improved wound healing using an argon DBD-based source called *P-Jet* [45]. To the best of our knowledge, this source has not been accredited as a medical device. For a novel, CE-marked, hand-held, and battery-driven plasma device called *PlasmaCare*, there is one recruiting interventional trial (ISRCTN98384076) with the primary outcome measure of a reduction of bacterial load as a basis for its prospective accreditation for wound healing. At the VU Medical Center Amsterdam, a phase I study (primary outcome: safety; secondary outcome: antimicrobial activity) using the *plasma* device for wound healing was recently completed (NCT03007264). A clinical trial on plasma-assisted wound healing after surgical removal of hemorrhoids (NCT03907306) is currently ongoing in the Russian Federation. Two trials to evaluate the efficacy and safety of the *RenewalNail* device (USA) targeting onychomycosis (fungal nail) were recently concluded (NCT03072550, NCT03216200). Another US-based device, the floating-electrode barrier discharge initially designed at Philadelphia-based Drexel University, is currently being tested by The Skin Center Dermatology Group in New York (NCT02759900) in patients with various skin disorders (actinic keratosis, acne, verruca plana (warts), and tinea corporis (superficial fungal infection)) up to the year 2023. The US-based Apyx Medical (formerly Bovie Medical Corp.) has completed a trial on their plasma device (J-plasma) for safety and effectiveness against facial wrinkles (NCT03286283).

Some of these niche applications are partially supported by clinical observations, for example, the decrease of the severity of atopic [46] and superinfected dermatitis [47] in patients. Future applications may concern treatment or pruritic disorders, leishmaniosis, erythema, fungal infections (especially onychomycosis), impetigo contagiosa, and folliculitis [48–50]. This is supported by numerous preclinical studies suggesting a microbicidal and antifungal action of plasmas, partially tested also on human skin [51–60]. Among the multiple applications of cold physical plasmas is their use in dentistry, where so far only one trial on dental restoration and caries prevention using the miniature atmospheric cold plasma brush (m-ACPB) has been completed (NCT01529606). Altogether, evidence for plasma-assisted wound decontamination and plasma-assisted wound healing based on (R)CTs is improving, although structured reviews are still missing. For other applications in dermatology, including the treatment of (pre)malignancies, RCTs are urgently warranted to increase the evidence level in plasma medical applications. The different plasma devices used across different countries will remain a drawback, each likely similar and dissimilar in several aspects at the same time. Here, basic and applied researches from physics to biology need to address the challenge of categorizing plasma sources and parameters under a unifying umbrella.

### 2.2. Oncology

Cancer is one of the biggest challenges in the medical field. Solely in 2018, it was responsible for almost 10 million deaths globally [61]. These striking numbers reveal the limitations of current therapy resources to improve overall survival and often also the patient's quality of life. For example, a challenge in the palliation of end-stage head-and-neck cancer patients is the extensive microbial growth on tumors, which produces a hostile odor and hampers social interaction. As these soft tumors are difficult to disinfect chemically, plasma was chosen for this purpose. While the decontamination procedure worked in all patients, tumor regression with plasma treatment was observed in some patients [62–65]. Another benefit was the healing of tumor wounds together with their decontamination with no or negligible side effects [62] and a decrease in the need for pain medication [63, 64]. These clinical results are important because they set the start point for future medical interventions with plasma, not only for palliation, but also for the treatment of less advanced cancers. However, treatment of metastatic lesions of malignant melanoma in end-stage patients with the plasma of the kINPen MED was so far of limited success [66]. Currently, one nonrandomized clinical trial (NCT03218436) in Tübingen, Germany is recruiting patients for the treatment of cervical intraepithelial neoplasia (ovarian cancer) with cold physical plasma.

A recent innovation in plasma oncology is the treatment of carcinoma *in situ*, e.g., actinic keratosis [67–69]. These dry, crusty, superficial lesions of the skin have a very high prevalence, and a significant percentage of lesions can develop into invasive squamous cell carcinoma over time. Patients with intraoral, precancerous leukoplakia or oral lichen planus lesions face a similar fate. Repetitive plasma treatment over several months successfully reduced and partially even removed these lesions [70]. Hence, plasma treatment may play a future role in the prevention of advanced cancer.

### 2.3. From Bench to Bedside to Bench

**Figure 2:**
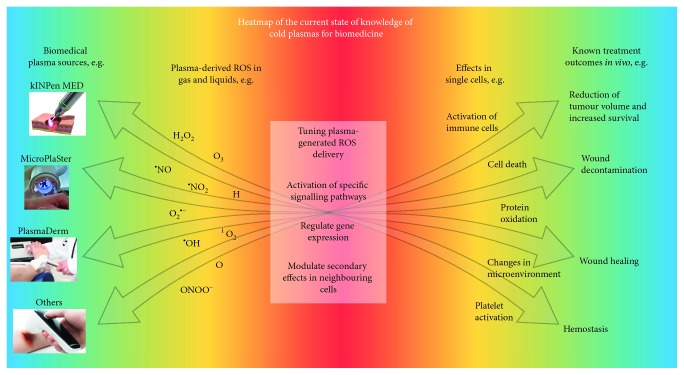
Heat map of the current state of knowledge of cold plasmas for biomedicine. Blue: known and well-characterized commercial plasma sources (left) and reported effects of plasma therapies in *in vivo* models and human patients (right). Yellow: many biologically relevant plasma-generated ROS in air or in liquids have been described (left); however, it is still a challenge to tune the setups to deliver specific ROS mixes for different biomedical applications. In the same way, multiple effects of plasma in cells have been reported, yet the mechanisms of action of plasma-generated ROS in cells has not been fully unraveled (right). Red: the current bottleneck in the field is the little information available on how to use plasma to activate specific signalling pathways and evoke a desired effect in cells to design better and more effective therapies.

Despite the clinical success of plasma treatment with some diseases, challenges remain. First, how can the rate of nonresponders seen in wound healing and cancer be decreased based on biological mechanisms yet to be identified? Second, how can new applications based on promising *in vitro* and *in vivo* research, e.g., treatment of metastatic melanoma, be implemented? Third, which are the promising therapeutic avenues in combining plasma treatment with existing therapies, e.g., immunotherapy in cancers, to maximize clinical outcome? These questions can be addressed in multiple ways, e.g., via tuning the chemistry of existing plasma sources, construction of novel plasma sources, finding the optimal dose and frequency of plasma treatment for each clinical application, and investigating promising combination therapies with plasma that seamlessly merge into existing clinical protocols. Thus, a number of iterations need to be tested in basic research on plasma redox chemistry and biomedicine to motivate and stratify therapeutic strategies in plasma medicine. Yet, while the physics of plasma is reasonably well explored, sufficient understanding in the chemistry and biology of plasma treatment is one current bottleneck in pinpointing best-practice plasma ROS patterns for the most efficient clinical response (Figure 2). Especially cell membranes, the key interface between plasma-derived ROS and cells, have been investigated only poorly so far. With plasma medicine being a field of unparalleled multidisciplinarity from physics and engineering, over chemistry and biology to medicine, the following sections provide the current working hypothesis in the field together with key knowledge gaps that need to be addressed to accelerate progress in this field.

## 3. Biological Mechanisms in Cells Exposed to Cold Physical Plasma

A macroscopic view of plasmas in biomedicine reveals multiple positive outcomes in patients treated with this technology. However, a microscopic view of the processes evoked by plasma in cells indicates that multiple mechanisms of action at the cellular and macromolecular levels are involved in exerting such effect, most of them being underexplored. In this section, we will discuss the collection of events that lead to the biological outcome previously described, considering the current state of the field with regard to challenges (Box 1) and opportunities (Box 2). Before discussing observations in plasma medical research, a brief summary of concepts in redox biology is given as a basis for plasma medicine.

### 3.1. Current Concepts in Redox Biology

Oxygen is a chemically aggressive molecule able to cause oxidative modifications in all biomolecules. At the same time, it is needed to preserve life in aerobic species. In order to prevent oxidative damage and maintain homeostasis, cells have developed efficient antioxidant mechanisms to cope with ROS produced by biological processes (i.e., mitochondrial respiration) and external insults (radiation, ionization). The misbalance between the levels of prooxidants and antioxidants in the cell results in oxidative stress, with the consequent accumulation of ROS and oxidative damage to the biomolecules that make up the cell. To prevent detrimental effects, cells are equipped with ROS detoxification mechanisms that can be enzymatic (catalases, peroxidases, and superoxide dismutases) and nonenzymatic (vitamin E, vitamin C, reduced glutathione, *β*-carotene, etc.). The outcome in redox biology will unequivocally depend on the type of ROS produced over a certain period of time at a specific location [71], as this is directly linked to the location and availability of the detoxification mechanisms to deal with the insult. The amount of ROS is also important, as low concentrations have different effects compared to higher concentrations, a phenomena coined as hormesis.

**Figure 3:**
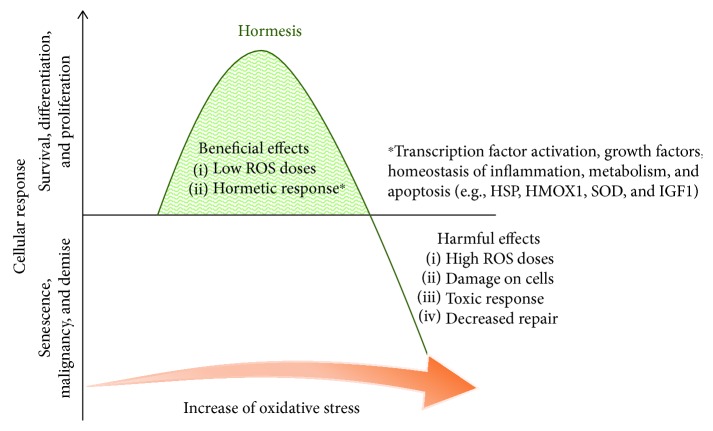
Scheme of hormetic responses. In the concept of hormesis, small concentrations of a given substance or molecules (including ROS) can have opposing effects between small and large concentrations.

Hormesis describes the biphasic dose response to an agent whereby a stimulatory or beneficial effect is obtained with a low dose and an inhibitory or toxic effect is achieved with a high dose. As an integral process of the normal function of cells, hormesis participates in multiple physiological processes that involve ion channels, enzymes, and transcription factors [72] (Figure 3). Hormesis then could be described as an adaptive response to environmental challenges in order to preserve homeostasis [73]. The biphasic dose response can be caused by multiple stimuli such as toxins, radiation, neurotransmitters, and ROS [74]. In wound healing and cancer, low concentrations of ROS have proproliferative effects, while high concentrations are deleterious [75–77]. Importantly, in both situations, signalling in response to ROS is key in subsequent biological effects.

ROS are constantly and purposefully made in the human body to exert a variety of responses. On the cellular level, ROS are produced to allow the development of oocytes after fertilization [78] and to attract neutrophils to the site of injury to clear pathogens and elicit inflammation [79]. On the molecular level, responses to ROS are related to both redox and phosphorylation signalling with proteins [80]. In the former, oxidases and reductases control disulfide bond formation of thiols, while in the latter, kinases and phosphatases control phosphor residues on target proteins. The binary states activate or inactivate the (binding) activity of proteins, and often both systems act in concert to achieve distinct biological responses. For instance, growth factor binding activates Src family members to phosphorylate peroxiredoxin 1 to render this antioxidant inactive. At the same time, NAPDH oxidase (NOX) is activated to produce superoxide in the extracellular space, which then dismutates to hydrogen peroxide, enters the cell through aquaporins, and reversibly oxidizes target molecules such as protein phosphatases [81]. At the same time, redox proteins also act as sensors of ROS. For example, upon ROS exposure, thioredoxin reversibly releases the apoptosis signal-regulated kinases (ASK1) to induce subsequent pathways for cell death [82].

With the exception of supraphysiological concentrations of ROS leading to immediate necrosis, ROS-mediated cell death is a form of regulated cell death as per consensus guideline [83]. This also delineates a link between ROS and a plethora of cell death pathways, including intrinsic apoptosis, ferroptosis, NETosis, lysosome-dependent cell death, mitochondrial pore transition-driven necrosis, parthanatos, necroptosis, and autophagy, largely because of the ROS' intrinsic and pleiotropic roles in metabolism, mitochondrial homeostasis, inflammation, and immunity. Importantly, not all types of cells can undergo all types of cell death. For instance, several tumor cell types are incapable of undergoing necroptosis [84], NETosis is primarily observed in myeloid cells [85], and oxycytosis is performed by red blood cells [86]. Attributing ROS- (and hence, plasma-) induced cell death to a certain modality is made complicated not only by the heterogeneous and cell-type-specific cell death responses but also by the fact that exogenous ROS exposure can also lead to quick endogenous ROS generation, making it difficult to distinguish primary from secondary ROS responses. Pinpointing the specific type of cell death is not only an academic question, as the type of cell death has important implications for the functional outcome in diseases. For instance, in wound healing, further excessive damage (e.g., necroptosis) may be discouraged for appropriate healing response, while in the treatment of tumors, a proinflammatory type of cell death would be encouraged to unleash the power of antitumor immunity.

### 3.2. Functional Consequences in Plasma-Treated Cells and Tissues

Hormesis accurately describes why plasmas are useful in both wound healing and cancer therapies: while the exposure to low levels of ROS can promote cell proliferation to support tissue regeneration, platelet activation, and blood coagulation [87–89], higher doses can induce cell death [90–92], endogenous ROS generation, and DNA damage, and lipid peroxidation [93]. This has been described in HaCaT cells exposed to plasma, where a low amount of ROS delivered over a minute of treatment was better tolerated than the fast delivery of the same amount of ROS over a few seconds [94]. Similarly, a study performed in ocular cells exposed to plasma for decontamination showed stimulatory effects at low doses and toxic effects at high doses [95]. It must be noted that the mechanisms involved in the hormetic response to ROS are differently activated (regarding type and strength) among tissues and cells, and therefore this should be considered in the analysis of the adaptive protective processes evoked by plasma [96].

One favorable advantage of cold plasma is the adjustable generation of biologically active factors, such as single or complex reactive species, at the site of interest by the admixture of water, oxygen, and/or nitrogen to argon gas [97–99]. As one consequence, cold plasma induces physical or chemical changes in fluids, cells, and tissues. The relatively short lifetime and the quick reaction between plasma-generated ROS and biomolecules, such as proteins, lipids, and nucleic acids, especially the short-lived species, lead to the formation of ROS intermediates. Such intermediates can directly function as signalling or redox-reactive molecules (e.g., NO and H_2_O_2_) in secondary reactions in biological environments [8, 100, 101]. Their high reactivity, diffusion, and delivery via pores, channels, and receptors influences the cellular availability and activates downstream signalling.

**Figure 4:**
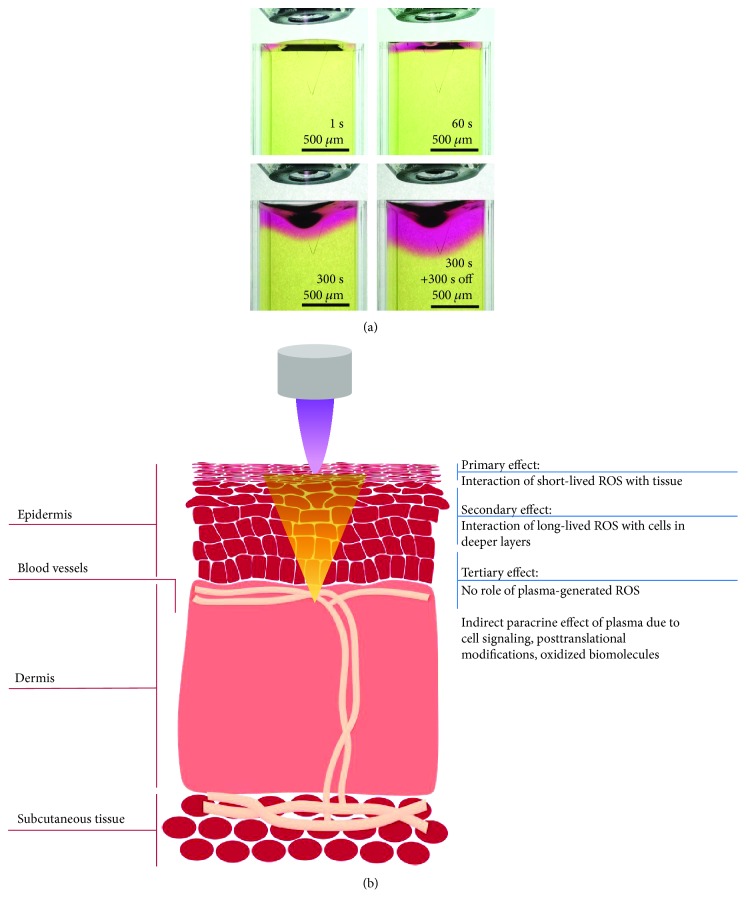
Models for the study of the penetration of plasma-generated ROS into tissue. (a) *In vitro* approach for the analysis of ROS penetration using 0.02% methyl red as a reporter of ROS in 0.5% agarose gel. The treatment applied with Ar/O_2_ (1%) kINPen MED at 4 mm distance demonstrates that the penetration depth is directly proportional to the treatment time (unpublished/original data). (b) Proposed mechanisms of action of plasma ROS and concomitant effects in tissues. The primary effect is exerted in the first layers of cells that directly interact with the short-lived ROS. At this level, oxidative damage is induced in the extracellular matrix, cell membranes, and intracellular components of cells located in the outermost region of the tissue. The long-lived ROS able to penetrate into deeper regions of the tissue elicit a secondary oxidative effect in cells. However, the effect of plasma extends to more profound regions of the tissue due to the oxidation of redox-sensitive cysteine and thiols in proteins with paracrine effects and via cell-to-cell communication.

The oxidizing properties of ROS have an important impact on membrane integrity [102, 103]. Reactive species oxidize hydrophilic head groups and lipophilic tails of the phospholipid bilayer, leading to an initial membrane rigidity and an increase in fluidity [104]. Although the penetration depth of plasma in tissues ranges from 5 to 40 *μ*m for O_3_ to a few millimeters for H_2_O_2_ and molecular oxygen (O_2_) [105, 106] (Table 1), the oxidizing nature of plasma by the oxidation of redox-sensitive cysteine and thiols in proteins [107–109] evokes paracrine effects [110, 111] and thereby changes of the microenvironment in deeper layers (Figure 4). Consequently, distant cells may benefit from cell-cell communication via paracrine mechanisms. One must also consider the presence of cells of the immune system, which are able to move across tissues and evoke a response at distant sites. Such is the case of immunogenic cell death (ICD), a mechanism proposed to mediate the effect of plasma in cancer and further discussed in this review. ICD-inducing therapies promote the expression of cell surface antigens and the release of damage-associated molecular patterns to activate cytotoxic T cells that kill the tumor cells and can stimulate antitumor immunity [112]. This mechanism is currently being studied in the field of plasma medicine [113], as it could extend the reach of plasma therapies from localized to systemic targets.

The maintenance of a physiological level of ROS is important for redox signalling [114–117]. An imbalance between the production and detoxification of reactive ROS intermediates affects the cellular stress level, e.g., cell cycle [118]. Cold plasma modulates numerous cellular processes related to redox signalling, and therefore, may be useful for targeting a plethora of specific, wound healing-related pathways.

### 3.3. Signalling Events in Wound Healing

**Figure 5:**
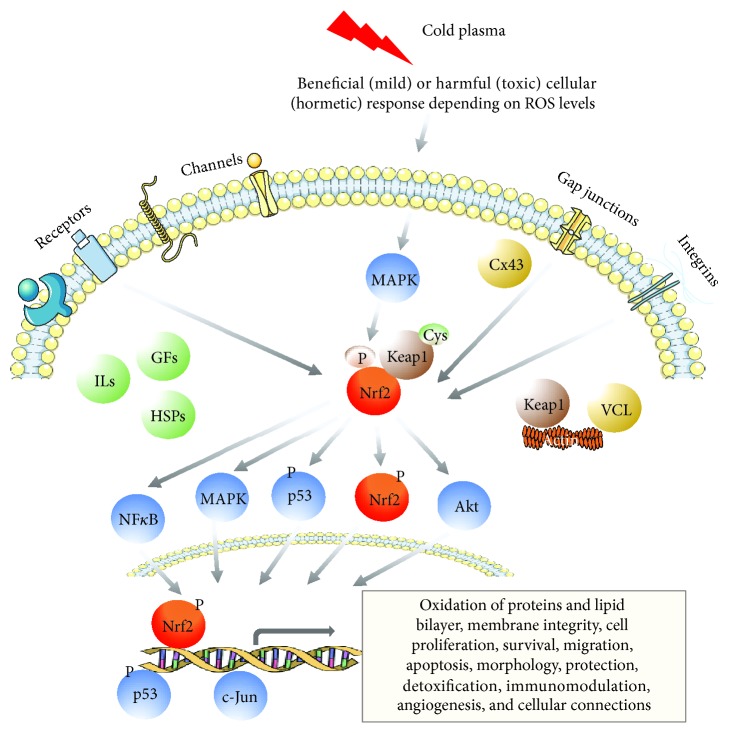
Overview of cold plasma-mediated signalling pathways, including oxidative stress (Nrf2), mitogen-activated protein (MAP) kinase, p53, Wnt/*β*-catenin, cytoskeletal, cell adhesion or growth factor (GF) signalling, and differentiation.

Changes in ROS levels trigger a coordinated action of redox-sensitive transcription factors (Figure 5) as part of cellular signalling (Table 2). Cold plasma significantly alters the nuclear factor erythroid 2-related factor 2 (Nrf2) pathway, as shown in global -*omics* analyses by microarrays, as well as by liquid chromatography and mass spectrometry, and in cytokine profiling [119–121]. In an immunocompetent murine wound model, gene and protein expression pattern revealed a strong regulation of specific targets of the Nrf2 pathway after a daily or three times per week treatment over 14 consecutive days [122, 123]. Nrf2 signalling, since its downstream targets act as sensors and/or effectors for increased oxidative stress, was ranked among the most active regulatory networks and canonical pathways after plasma treatment. Nrf2, itself, activates cellular rescue pathways against oxidative injury, inflammation, or apoptosis and functions in cellular defense against imbalances in redox homeostasis [124, 125]. The primary event in downstream signalling of Nrf2 is the recognition of plasma-generated ROS by specific oxidative stress sensors such as the actin-binding protein Kelch-like ECH-associated protein 1 (Keap1) [126]. Under basal conditions, Nrf2 is associated with Keap1. This vital factor in Nrf2 signalling cascade retains Nrf2 in the cytoplasm where Nrf2 is targeted for ubiquitin-mediated degradation [127, 128]. After the release of Nrf2 from Keap1 by oxidation events at cysteine, Nrf2 translocates to the nucleus, binds to antioxidant responsive elements (AREs) that are located in the promoters of its target genes, and activates their transcription [120, 123]. To scavenge ROS and inhibit oxidative damages, cells activate Nrf2 and its downstream genes, which encode ROS-detoxifying enzymes and antioxidant proteins. Among the most robustly increased proteins, heme oxygenase 1 (HO-1), NADPH quinone oxidoreductase 1 (Nqo1), carbonyl reductase 1 (Crb1), *γ*-glutamylcysteine ligase catalytic (GCLC) and modifier subunit (GCLM), superoxide dismutases 1-3 (Sod1-3), thioredoxin (TRx), catalase (Cat), glutathione peroxidase (GPx), cytochrome P450, and nonenzymatic antioxidants like glutathione were found. Proteins involved in thiol group reduction or coupling (glutathione-S-transferases, e.g., GstK1, GstO1, and GstP1) showed an increased abundance (ca. 70 molecules), demonstrating that the glutathione metabolism is affected, which is a marker for an Nrf2-related signalling event. The strongly increased abundance of heat shock proteins (Hsp90 and Hsp40 derivatives) also indicates cellular response to plasma in terms of thermal or chemical stress [121].

Morphological changes such as cell size [122], the reorganization of cytoskeleton, and altered cytoskeletal [129, 130] and adhesion molecule expression [131–133] are indispensable for skin repair in wounds and in the metastatic behavior of cancer cells. Plasma-generated ROS alter the barrier function and intercellular communication such as gap junctional protein expression by a transient blocking of connexin 43 (Cx43) [122] and a modulation of tight junctional zona-occludens protein 1 (ZO-1) in skin cells [134, 135]. The formation and maintenance of the skin barrier function largely depends on the regulation of these cellular connections (e.g., adherence and tight and/or gap junctions), expression of junctional proteins, surface markers, and growth factor receptors [136]. Also, wound healing requires a well-balanced expression of extracellular matrix (ECM) and matrix metalloproteinases (MMPs) [137, 138]. In this regard, chemical modifications of ECM and MMPs were shown, affecting cells and tissues by cold plasma-generated ROS [139, 140]. However, transepidermal water loss (TEWL) was only transiently reduced after plasma treatment but not further affected in the course of time [141].

Beyond the regulation of antioxidant gene expression, Nrf2 also contributes to the anti-inflammatory process by orchestrating cytokine secretion of pro- and anti-inflammatory factors, and an early infiltration and recruitment of inflammatory cells such as macrophages [142]. The regulation of most of such events, including inflammation and immune cell infiltration [123, 143, 144], depolarization of macrophages [145, 146], mitochondrial function and content [147], angiogenesis (e.g., Akt) [110, 123, 148], growth factor signalling [123, 149], and cellular viability [134, 150] are further responses after plasma treatment. Studies combining electrical fields with plasma treatment demonstrated a synergistic metabolic activation of mammalian cells [151] besides the antibacterial effect [152]. Moreover, plasma-induced activation of Nrf2 accelerates wound healing and provides a faster wound closure by a concomitant increase in basal proliferation and cellular migration [122, 153]. A rapid and transient activation of the proliferative-acting extracellular signal-related kinase ERK1/2, and a slower but sustained activation of stress-activated p38 and c-Jun N-terminal kinases was detected in skin cells [119, 154].

Beside this proliferative effect, apoptotic events include the removal of inflammatory cells and inhibition of scar formation of granulation tissue at later stages of wound healing. The lower frequency of TUNEL-positive apoptotic cells on early time points in plasma-treated wounds, either due to enhanced macrophage numbers and activity or a redox-mediated suppression caused by plasma-derived ROS intermediates, and the increasing number of TUNEL-positive apoptotic cells at later time points is an essential prerequisite in skin wound healing [123]. Redox-sensitive transcription factors, such as the tumor suppressor protein p53, are susceptible to ROS-dependent modifications, which could impact their biological functions and activities [155]. Moreover, p53 can mediate a two-phase Nrf2 response: when p53 expression is relatively low, p53 enhances the protein level of Nrf2 and its target genes to promote cellular protection and survival at basal levels in a p21-dependent manner [156]. Contrary, the Nrf2-mediated survival response is inhibited and senescence/apoptosis at higher ROS levels is supported in the repression phase [157]. This cross-talk between oxidative stress (Nrf2 signalling) and DNA damage (p53 activation) defines the critical point where cell injury may switch from an adaptation to an injury state [158]. Additionally, the phosphorylation status and therefore the activity of p53 depends on wound stages and is timely regulated [159, 160]. A transient inhibition of p53 supports the early cell proliferation required [157]. Later apoptotic events are induced via caspase activation [119, 154], cell-cycle disruption [161], and other multiple pathways [162, 163]. Cold plasma transiently enhances total p53 protein expression, induces nuclear translocation of p53, and alters the phosphorylation level of p53 in a treatment and incubation time-dependent manner [164]. Findings further suggested plasma-induced cell reactions of stress sensing, along with metabolic alterations [143, 165]. The interaction with the signal transduction pathway of p53 and related processes fosters the understanding of plasma-induced cell protection against DNA damage or DNA strand breaks.

### 3.4. Effects on Cancer Cells

Plasma therapies for cancer have shown promising results in multiple cancer types using a variety of plasma sources [166]. Most studies report a decrease in cell viability and elevated cytotoxicity upon plasma treatments [167–177]. Part of the damage is induced to the cell membrane, the first barrier to deal with the oxidative stress induced by plasma. The first effect observed in plasma-treated cancer cells is lipid peroxidation, a process where lipids with carbon-carbon double bounds such as glycolipids, phospholipids, and cholesterol are oxidized [178]. The extensive peroxidation of lipids upon plasma treatment, if present, may increase the entropy in the plasma membrane and alter the assembly, dynamics, and structure of lipids, facilitating pore formation [104, 179, 180]. In fact, the highly porous, disorganized plasma membrane serves as the entry door of multiple extracellular ROS, a process observed in necrotic cells [181]. Interestingly, lipid peroxidation is characteristic of ferroptosis, a Fe(II)-dependent cell death mechanism driven by oxidative stress and consecutive lipid peroxidation [182]. One report suggests that plasma treatment could promote ferroptosis in cancer cells via the reduction of Fe(III) to Fe(II) stored in ferritin [183]. In this case, the increase in Fe(II) available within the cancer cell could contribute to the Fenton reaction and the consequent formation of the highly reactive ^•^OH radical, able to react with any biomolecule present at close proximity [184].

Cancer cells are more sensitive than normal cells to oxidative stress due to the increased steady-state ROS levels produced. The high glucose uptake and transformation to lactate, even in the presence of oxygen (also known as the Warburg effect), is responsible for the accumulation of intracellular ROS in cancer cells [185]. It has been suggested that increasing the oxidative stress by exogenous ROS (such as plasma treatments) to a threshold incompatible with cell viability could selectively eliminate cancer cells without damaging the healthy ones [186, 187]. In the plasma field, it has been suggested that an increase in aquaporins [188] or a decrease of cholesterol in the plasma membrane of cancer cells [179, 189] facilitates the transport and permeation of ROS to the intracellular compartment, supporting a selective effect of plasma on cancer over normal cells. The latter may also be mediated by cell-cycle arrest [190]. It is possible that the combination of these factors favors the selective elimination of cancer cells by plasma.

Plasma therapies for cancer have shown positive results both for localized and metastatic cancers in animal models, especially in melanoma [191]. Plasma can also induce immunogenic cell death (ICD), a regulated cell death mechanism that involves the release of damage-associated molecular patterns by cancer cells and the recruitment of immune cells to eliminate the tumor [83]. Direct plasma treatment of glioblastoma xenografts has been shown to increase the survival rate and reduce tumor volume [192], as well as to induce apoptosis and cell-cycle arrest [193]. This in turn may increase their sensitivity to common chemotherapeutic drugs such as gemcitabine [194, 195], doxorubicin [196], and novel mitochondrial complex IV [197], as well as HSP90 inhibitors [198] as well as to traditional radiotherapy [199]. Interestingly, plasma treatments could suppress the growth of irradiated and nonirradiated remote melanoma tumors in mice (known as abscopal effect), suggesting the participation of the innate immunity in the response to treatment [200]. The antiproliferative effect observed in plasma-treated tumors equally affects chemoresistant and chemosensitive cancer cells [201]. Plasma-treated solutions have proved to be effective against metastatic cancers in murine models. Intraperitoneal injections of plasma-treated medium were able to inhibit dissemination of ovarian cancer [202], and plasma-treated medium and saline solutions reduced the tumor burden, promoted the infiltration of macrophages, and increased T cell activation as well as immunogenic cancer cell death *in vivo* [203–205]. With direct plasma treatment, ICD can be induced in localized colorectal tumors [206] and melanoma tumors in mice by the short-lived species produced by plasma [207]. Whether plasma-induced ER stress [208] links to plasma as a type I or type II ICD inducer [209] is the subject of current investigations. To date, there is no report of resistance to plasma treatment, suggesting that plasma could be a promising therapy for cancer.

## 4. Cellular Membranes as a Link between Plasma Chemistry and Biology

**Figure 6:**
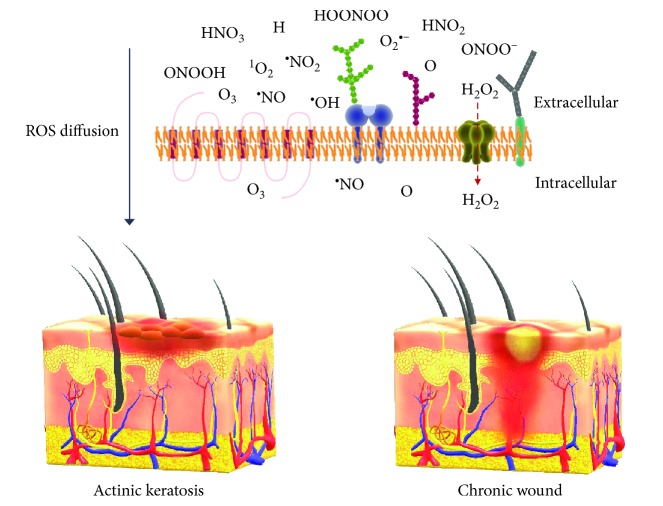
The cell membrane is the key compartment that plasma-derived ROS need to penetrate or interact with to elicit biological responses. While some ROS are able to penetrate cellular membranes (e.g., ozone, nitric oxide and atomic oxygen), other more polar ROS cannot (e.g., singlet delta oxygen, nitrite, hydroxyl radical, superoxide anion, hydrogen, and peroxynitrite). Hydrogen peroxide is actively transported into the cells via transporters such as aquaporins.

One way for plasma treatments to be effective is that plasma-derived ROS cross or interfere with the cell membrane to affect its stability and permeability, ultimately altering the intracellular circuitry [210]. The field of redox biology has extensively addressed the effect of ROS in cell membranes; for that reason, this section will put the effects of plasma treatments on cell membrane components in context with the current knowledge in redox biology (Figure 6). Several studies have already provided evidence that skin lipids from human volunteers undergo oxidative changes upon plasma treatment, although the functional consequences remain elusive [211–214].

### 4.1. Cellular Membranes as a Target, ROS Source, and Transporter of Plasma-Derived ROS

Those ROS and RNS produced by plasma in the gas phase that are able to penetrate the liquid or soft interphase characteristic of biological substrates may directly or after transformation into additional ROS, react with cellular molecules and the extracellular matrix. The exterior of mammalian cells is composed of a complex lipid bilayer with a highly variable and dynamic chemical composition, additionally diversified by intercalated proteins (compiled in [215]). Due to their projected position and chemical nature, lipids represent “ideal” targets for oxidative modifications by plasma-derived ROS. Lipids comprise a chemically heterogeneous group of compounds that often combine hydrophilic and lipophilic substructures in the molecule [216]. In phospholipids, long-chain fatty acids are connected via a polyalcohol bridge (e.g., glycerol) to a polar head group consisting of an orthophosphate residue and an amine (choline, ethanolamine), creating a zwitterion. Various numbers of isolated double bonds are frequently found in the fatty acid tails, increasing sensitivity towards oxidative events. Attacking the weak sp^1^ carbon-hydrogen bond at the allyl position easily yields hydroperoxyls, hydroxylations, and radical intermediates. Subsequent reactions, like the Hock rearrangement may lead to chain breakage [217]. The resulting short-chained fatty aldehydes like 4-hydroxynonenal are relevant second messengers (see Section 4.2), and the residual aldehyde fatty acids are more polar, decreasing the order and crystallinity of the membrane [178]. Further addition or substitution reactions can occur at the double bond(s), yielding nitro- or chlorohydroxy fatty acids, depending on the attacking species [218, 219]. Accordingly, lipids are common targets of oxidative modifications by plasma-derived ROS and/or RCS (reactive chlorine species) that occur in specific conditions. Maheux et al. investigated the impact of a helium/nitrogen-driven DBD jet onto liposomes made of 1,2-di-(9Z-octadecenoyl)-sn-glycero-3-phosphocholine (DOPC) [220]. Significant changes to the physical properties of the lipid particles, including size and zeta potential, were accompanied by the detection of dioxidized DOPC and chlorohydrins. Yusupov et al. revealed the impact of plasma-derived species, especially the ^•^OH radical, on lipids and lipid complexes, e.g., bilayer models, using atomic scale simulations. Taking lipid bilayer geometry, radical species half-life, and reactivity into account, the predominant target was identified as the lipid's head group. In contrast, a strong impact on the fatty acid chain yielding cleavages was observed experimentally. A number of not fully resolved structures connected to the investigated lipid but showing cyclisation in the head group suggested that a direct interaction of short-lived species, especially ^•^OH radicals, with the head groups cannot be excluded and may have contributed to the side-chain oxidation. Ultimately, the sum of oxidations yielded a decreased membrane stiffness of the model liposomes [104].

Plasma treatments have been shown to increase the cell membrane permeability [221, 222]. Further, ROS delivered by plasma such as O_2_, HOCl, O_3_, ^1^O_2_, ^•^NO, and ONOO^−^ can trigger radical chain reactions, resulting in propagated lipid oxidation [223, 224]. The superoxide anion radical O_2_^•-^, produced either by plasma and/or as a cellular product from a single-electron transfer reaction, is relatively nonreactive by itself. However, its reaction with NO yields the strong oxidant peroxynitrite, which in turn contributes to lipid oxidation. Extracellular O_2_^•-^ and NO can be produced from physical plasma as well as certain types of cells as a basis for peroxynitrite generation [225]. The accumulation of oxidized lipids in the bilayer upon plasma treatment reduces the electric field threshold required for pore formation and decreases the mechanical strength, thereby increasing the permeability and fluidity of the membrane [179, 180, 226]. Similarly, lipid oxidations have been proposed to occur during the electroporation of cells to facilitate membrane permeability [180]. This suggests a concomitance of both processes and emphasizes that lipid oxidation and/or chain cleavage are key factors determining membrane fluidity and polarity and ultimately membrane penetration. Of note, the membrane lipid composition of normal and cancer cells differ in the reflection of their metabolic state, contributing to a certain graduation of the impact of plasma or other prooxidant physical treatment regimens [227, 228].

### 4.2. Secondary Messengers Deriving from ROS or Plasma-Derived ROS

When looking into singular lipid structures and related functional consequences in biological systems, a vast list could be compiled. Many lipid oxidation products act as second messengers having almost unrestricted access due to their ambipolarity. Well-known examples are the fatty acid oxidation derived eicosanoids with extensive impact in inflammation regulation that are also targeted by mass-market and high-selling drugs [229]. The first step, the enzymatic release of arachidic acid from a phospholipid can be achieved by plasma as well, thereby increasing the pool for the cyclooxygenases performing the following two-stage oxidation leading to the intermediate prostaglandin H_2_. It contains an endoperoxide, a structure that can be derived from a singlet oxygen, a common species in plasma. Downstream, this endoperoxide is replaced by oxo- and hydroxyl groups. Although these structures are complex, many steps can be performed by the plasma, opening an avenue to modulate a range of pathways, including inflammation, cardiovascular effects, or pain perception. Interestingly, a decrease of pain was repeatedly reported by patients undergoing plasma treatment of chronic wounds (see the results reported in [39]).

Some lipid oxidation products are cytotoxic and can induce apoptosis, such as 7*α*,*β*-hydroxy-, 7-oxo-, and 5,6-epoxycholesterol produced from oxidized cholesterol [230]. The reaction of ^•^OH with cholesterol can lead to the formation hydroperoxyl radicals (HO_2_^•^) and the corresponding superoxide anion radicals (^•^O_2_^−^), important due to their multiple effects in cells. Excess HO_2_^•^/^•^O_2_^−^ disproportionate spontaneously or is enzymatically reduced forming H_2_O_2_, ultimately yielding again ^•^OH radicals through Fenton or Haber-Weiss reactions, potentially leading to the initiation of the chain oxidation of (poly-) unsaturated phospholipids [178]. In the skin, plasma-derived H_2_O_2_ and O_2_ have been named the main ROS responsible for cholesterol oxidation [231]. It is possible that the propagation of the reaction continues within the plasma membrane, as O_2_ concentrates close to the lipid tails inside the lipid bilayer where it can oxidize other lipids [189, 231]. Interestingly, ^1^O_2_ can also oxidize cholesterol to produce 5*α*-OOH, the most damaging hydroperoxide product due to its ability to accumulate and to migrate from the production point to more sensitive sites where iron-mediated cytotoxicity can be induced [224]. However, the participation of 5*α*-OOH in the response to plasma treatments is so far unknown. Other ROS-derived lipid peroxidation products such as 4-hydroxynonenal (HNE) can form DNA adducts [232]. HNE in particular is an important second messenger molecule that participates in the activation of Nrf2, a regulator of cellular resistance to oxidants [126].

Oxidized phospholipids (OxPL) can also serve as ligands in damaged or stressed cells that are recognized by receptors in cells of the innate immune system [233]. The scavenger receptors CD36, SRA, and SRB1 (present in anti-inflammatory M2 macrophages) bind to OxPL in apoptotic cells to trigger their clearance by the immune system [234]. Plasma has been shown to effectively induce apoptosis in cancer cells [235–238], and it is possible that OxPL was formed in their plasma membranes. Interestingly, it has been shown that plasma favors monocyte differentiation towards a M2-like macrophage profile accompanied by an increased CD36 expression [145]. It is conceivable to think that plasma treatments could participate in both the induction of apoptosis in cancer cells and their clearance by macrophages. Nitrogen dioxide (NO_2_) generated from peroxynitrite can originate nitrofatty acids (NO_2_-FAs) [239] that can inhibit the propagation of lipid peroxidation and protein nitration and therefore counteract the proinflammatory and cytotoxic effects [240]. NO_2_-FAs can release ^•^NO into the cell, inhibit the activation of the transcription factor NF*κ*B, and alter the activity of proteins involved in antioxidant responses [188]. It has been shown that a plasma-treated medium attenuated the NF*κ*B pathway in the MDAMB231 human breast adenocarcinoma cell line [241], and the direct plasma treatment combined with cetuximab modulated the NF*κ*B and p53 signalling pathways in head-and-neck cancer cells [242]. In the same way, plasma decreased the antioxidant activity of glioblastoma, thyroid carcinoma, oral carcinoma, and nonmalignant embryonic cells [243], which suggests a possible participation of NO_2_ and NO_2_-FAs in the responses observed. Further studies of these intermediates and signalling pathways involved in the response in the context of plasma therapies should be done.

### 4.3. Impact of Plasma-Derived ROS on Membrane-Associated Proteins

Beside lipids as the dominant compounds in a cell membrane, numerous proteins are integrated into it. As discussed, ROS can be actively transported into the intracellular compartment (aquaporins) or neutralized by enzymes such as catalase or superoxide dismutase, thereby modulating the impact of plasma. The expression of these proteins in the membrane determine the susceptibly of cells towards plasma. These proteins are also susceptible to oxidation by exogenous ROS. Their main targets are amino acids with aromatic side chains [244] and those containing sulfhydryl groups [245]. The reaction of plasma-derived ^•^NO and O_2_^•-^ yields the strong oxidant peroxynitrite/peroxynitrous acid (ONOO^−^/ONOOH) which reacts with lipid hydroperoxides to form ^1^O_2_ and induce protein oxidation [178]. ROS can induce functional and structural changes in cell membrane proteins that result in their activation, change in gene expression levels, or degradation, as observed in cells treated with plasma (Table 3).

Although ROS can exert negative effects in cells, H_2_O_2_ is normally produced extracellularly in low concentrations to serve in both autocrine and paracrine fashion [246]. NOX present in the cell membrane generates O_2_^•-^ into the outer cell environment, which is later dismutated into H_2_O_2_ [247]. The main role of H_2_O_2_ as a signalling molecule is to oxidize proteins on specific sites to modulate their function and therefore regulate gene transcription, proliferation, metabolism, and migration [248]. Because the diffusion of H_2_O_2_ across membranes is limited, aquaporins (AQPs) transport H_2_O_2_ into the intracellular space to meet the physiological demands [249]. It has been reported that cancer cells overexpress aquaporins in their cell membrane compared to normal cells, which could favor H_2_O_2_ transport into the cytosol [250, 251]. This particular feature of cancer cells could explain the selective effect of plasma in cancer cells, as described before [188]. The contribution of aquaporins to the response to plasma-generated ROS is currently under study, as only the role of AQP1, AQP8, and AQP9 in H_2_O_2_ transport upon plasma treatment has been reported (Table 3). Cancer cells that are resistant to ROS-induced apoptosis can overcome the cytotoxic activity of exogenous H_2_O_2_ by presenting catalase in the outer layer of the cell membrane [252]. Membrane-bound catalase decomposes H_2_O_2_ and ONOO^−^ and oxidizes NO present outside the cells. Catalase therefore interferes with the ROS signalling through the HOCl and the NO/ONOO^−^ pathway [247, 253]. Interestingly, ^1^O_2_ produced during the exposure of cancer cells to the plasma-treated medium has been shown to inactivate the enzymatic activity of membrane-bound catalase, restoring the activation of the apoptotic pathway [254].

## 5. Conclusion

Treatment with cold physical plasma-derived ROS provides new therapeutic avenues in the therapy of a number of diseases. While the composition of ROS in the plasma gas phase, as well as the functional consequences in cells, is reasonably well explored, much more effort is needed to explore in greater detail the interphase reactions between the ROS cocktail and cell membranes and tissues. To accelerate such research, novel tools for studying the effects of different kinds of ROS, as well as consensus guidelines of the plasma medicine community, will be of great benefit.

## Figures and Tables

**Box 1:**
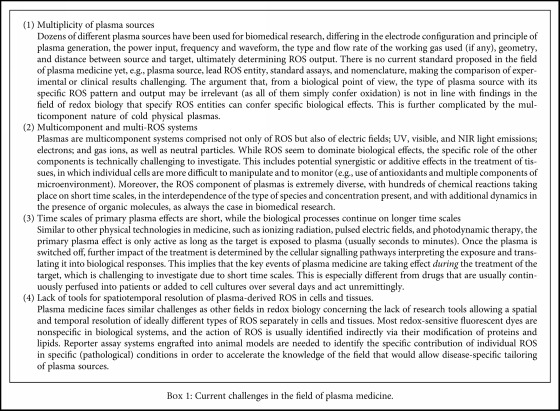
Current challenges in the field of plasma medicine.

**Box 2:**
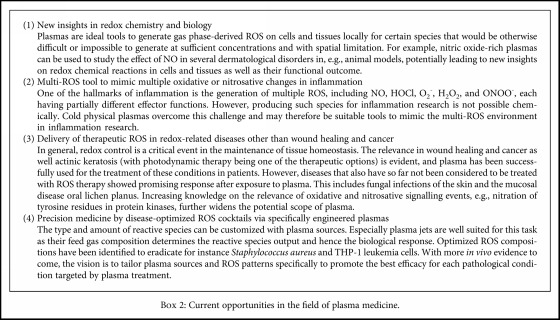
Current opportunities in the field of plasma medicine.

**Table 1:** Overview of reported studies on penetration depths of plasma-derived ROS in original and artificial tissue models.

Penetration depth	Plasma treatment	Tissue or biosurface studied	References
*In vivo* models			
10 *μ*m	kINPen09	Human skin	[255]
36.8 ± 14.2 *μ*m	kINPen09	*In ovo* tumour of pancreatic adenocarcinoma cells	[106]
~65 *μ*m^∗^	MicroPlaSter *β* plasma torch system	Skin wounds in 129 Sv/Ev female mice	[110]
2.8 mm	Helium plasma jet	Bladder carcinoma tumors in BALB/c nu/nu male mice	[256]
~50 *μ*m^∗^	Atmospheric-pressure helium plasma jet	Skin of BALB/c female mice	[257]
~300–400 *μ*m	kINPen09	Hair follicles	[60]

*In vitro* surrogate models for real tissues			
1 mm	Helium plasma jet	ROS delivery through pig skin into liquid	[256]
500–1500 *μ*m	Helium+0.5% O_2_ plasma jet	ROS delivery through pig muscle into various liquids	[258]
100–470 *μ*m	Helium+0.5% O_2_ plasma jet	KI starch-containing gelatin films	[259]
150 *μ*m	Helium plasma jet	2,7-Dichlorodihydrofluorescein/gelatin model	[260]
150 *μ*m	Helium plasma jet	ROS sensor-containing phospholipid vesicles in gelatin	[261]
1 mm	Helium linear- and cross-field plasma jets	ROS delivery through gelatin or gelatin+NaNO_2_ films into distilled water	[262]
1 mm	Helium plasma jet	ROS delivery through gelatin, gelatin+BSA, or poly(vinyl alcohol) targets into various liquids	[263, 264]
6 mm (6 min) 8 mm (36 min) 11 mm (66 min)	Argon plasma jet	KI starch gel	[265]
2 mm (36 min) 4 mm (66 min)		2% agarose	
1.5–5.8 mm	Low-temperature plasma jet	ROS delivery through agarose films into liquid	[256, 266–269]
1–2 mm	Helium plasma jet	Agarose films	[270, 271]
2 mm	Helium plasma jet	DNA damage in HEPES solution, phospholipid vesicles, or DNA embedded in gelatin	[272]

*In silico* models			
Plasma ROS: 10–20 *μ*m H_2_O_2_, O_2_^−^: 1–1.2 mm HO_2_: 20–250 *μ*m O_3_: 5–40 *μ*m	Low-power He-O_2_ plasma	Highly hydrated biofilms and plasma-tissue interaction models	[273]

^∗^Retrospectively measured with software from published images.

**Table 2:** Overview of cold plasma-mediated signalling pathways, including oxidative stress (Nrf2), mitogen-activated protein (MAP) kinase, p53, Wnt/*β*-catenin, cytoskeletal, cell adhesion, or growth factor signalling and differentiation. He-GIW: helium-guided ionization wave; SMD: surface microdischarge.

Signalling	Cell type(s)	Plasma source	References
Nrf2	Keratinocytes (HaCaT)	He-GIW	[140]
	THP-1 monocytes (human)	kINPen	[274, 275]
	Breast, pancreatic, colon cancer, and melanoma	kINPen	[276]
	Osteosarcoma cells	kINPen	[277]
	Periodontal ligament (PDL) cells	Plasma one dental	[278]
	Rat skin cells	Single-jet system	[279]
	Murine skin cells	kINPen	[123]
	Keratinocytes (HaCaT)	kINPen	[120, 121, 134, 148]
	T-lymphoblastoid leukemia cells	DBD	[236, 280]

NF*κ*B, MAPK	Monocytes, THP-1, and Jurkat	kINPen	[119, 154]
	Cancer cells	DBD	[281]
	HNC cells	Spray-type jet	[282]
	Cancer cells (G631)	APPJ	[283]
	Cancer cells (ES2)	NEAPP	[202]
	Keratinocytes (HaCaT)	kINPen	[284]
	Cancer cells (A375, 875)	Surface BD	[285]

p53	Melanoma cells	SMD	[286]
	Keratinocytes (HaCaT)	DBD	[287]
	Cancer cells	Different	[166]
	Cancer cells (HSC3)	DBD oxygen	[288]
	Cancer cells	DBD	[281]
	T98G, A549, HEK293, and MRC5	Soft plasma jet	[289]
	Periodontal ligament (PDL) cells	Plasma one	[278]
	Melanocyte cancer cells	APPJ	[283]
	Keratinocytes (HaCaT)	kINPen	[164]
	Murine skin cells	kINPen	[123]
	Cancer cells (Huh7, Alexander, and HepG2)	Air based	[290]
	Keratinocytes (HaCaT)	DBD	[291]
	T-lymphoblastoid leukemia cells	DBD	[236]

Wnt/*β*-catenin, cell adhesion	Melanoma cells (SK-Mel-28)	kINPen	[292]
	Keratinocytes (HaCaT)	DBD	[293, 294]
	Keratinocytes (HaCaT)	DBD	[131, 132, 295, 296]
	MNC	DBD	[297]
	Normal and cancer cells	Jet	[133]

Cytoskeletal	Skin cells	DBD, kINPen	[110, 153, 298]
	Keratinocytes (HaCaT)	DBD	[287]
	Cancer cells (BHP10, TPC1)	Spray-type jet	[299]
	Human dermal fibroblasts	Jet like	[129, 130]
	Skin cells (HaCaT, MRC5), melanoma cells	kINPen	[122, 292]

Differentiation growth factors	Neuroblastoma 2a (N2a)	DBD	[300]
	Keratinocytes (HaCaT)	kINPen	[149]
	Human 3D skin model	Single jet (MEF)	[301]

**Table 3:** Overview of the main components of the cell membrane and their role in the response to plasma treatment.

Molecule	Key physiological role(s)	Reported role in response to plasma	Redox-mediated downstream effects
Transporters			
AQP1	Water, H_2_O_2_ [302], CO_2_, NO, and ammonia	Favored H_2_O_2_ permeation into intracellular compartment [251]	Signalling via the Keap1/Nrf2 system [303]
AQP3	Water, urea, H_2_O_2_ [304], glycerol, and ammonia. Involved in cell proliferation, invasion, and angiogenesis [305]	Unknown	Activation of the Nox-2 and PI3K/Akt or MAPK pathway [306]
AQP5	Water and H_2_O_2_ [307]. Involved in tumor formation, cell proliferation, and migration [308]	Unknown	Role in tumor formation related to its phosphorylation status [309]
AQP8	Water, H_2_O_2_ [310], and ammonia	Required for anticancer effect of plasma-treated medium (PTM) on glioblastoma cells [311]	EGF induces AQP8 expression via EGF/EGFR-ERK1/2 pathway [312]. H_2_O_2_ transport is controlled by redox-mediated modifications [313]
AQP9	Water, H_2_O_2_ [314], urea, glycerol, lactate, and pyruvate [309] AQP9 knockdown reduced H_2_O_2_-induced cytotoxicity [314]	Its absence does not impair H_2_O_2_ transport upon treatment with PTM in glioblastoma cells [311]	Target of protein kinase A [307]. Possible interaction with ERK1/2 and MMP9 to enhance invasion and migration of prostate cancer cells [308]

Cell membrane receptors			
Epidermal growth factor receptor (EGFR)	Receptor tyrosine kinase involved in signal transduction to stimulate proliferation and cellular growth and block apoptosis	EGFR was degraded and dysfunctional in EGFR-overexpressing oral squamous carcinoma after plasma treatment [315, 316]	Moderate exogenous H_2_O_2_ induces the redox activation of EGRF and increases protein kinase activity [317].
Transient receptor proteins (TRP)	Calcium-permeable and voltage-independent cation channels which act as multimodal sensors of external stimuli	Unknown	In response to oxidative stress, TRPC3 and TRPC4 increase the intracellular Ca^2+^ concentration that leads to cell death [318]
Integrins	Responsible for cell-to-matrix and cell-to-cell adhesion. Integrins transduce the external signals to the cytoskeleton	DBD/air plasma enhanced expression of *α*_2_-integrin/CD49b and *β*_1_-integrin/CD29 in HaCaT cells [295] Marginal decrease in *α*5- and *β*_1_-integrins in primary fibroblasts and PAM cells [319] Plasma activates *β*_1_-integrins on the cell surface of WTDF3 mouse fibroblasts [320] kINPen plasma jet treatment downregulates integrin expression in MRC5 cells [122] and increases *β*_1_-integrin in HaCaT cells [132]	Integrin-linked kinase (ILK) signalling via PKB/Akt can suppress apoptosis and anoikis [321]. ILK is required to maintain redox balance [322] NRF2-mediated oxidative stress response
E-cadherin	Calcium-dependent cell-to-cell adhesion receptor	kINPen plasma jet treatment decreases E-cadherin expression in HaCaT cells [122, 132] Argon plasma modulates E-cadherin function and induces its internalization in HaCaT cells *in vitro* and decreases the amount of E-cadherin in mice epidermis [323]. Others report an increase in E-cadherin expression in the wounds of rats [324]	Oxidative stress causes the selective disruption of E-cadherin and beta-catenin cell adhesion complexes [325] In response to oxidative stress, E-cadherin binds to Nrf2 to restrain Nrf2 nuclear localization and activity [326] Assembly of E-cadherin activates several small GTPases and, in turn, the activated small GTPases control the effects of E-cadherin-mediated adhesions on epithelial biogenesis [327] Involvement of ROS in the regulation of cell adhesion and signal transduction functions of integrins and cadherins, pointing to ROS as emerging strong candidates for modulating the molecular cross-talk between cell-matrix and cell-cell adhesion receptors [328] Redox-regulation of EMT [329]
Focal adhesions	Adhesive contact that anchors the cell to the extracellular matrix that mediates mechanical and biochemical signalling	Plasma increased the amount of vinculin and the focal adhesion size in WTDF3 mouse fibroblasts [320]	Oxidative stress activates focal adhesion kinase by Src kinase- and PI3 kinase-dependent mechanisms, which accelerates cell migration [330]

Lipids			
Cholesterol	Provides rigidity to the cell membrane and controls membrane fluidity [331]	When present at low concentrations in the cell membrane, plasma oxidation facilitates pore formation and passing of ROS [179]. Unknown effect of toxic by-product 5*α*-OOH after plasma treatment	Oxidation by-products such as HO^•^_2_ can generate intracellular H_2_O_2_ and ^•^OH, and propagate lipid oxidation [178]. Induction of apoptosis by 7*α*,*β*-hydroxy-, 7-oxo-, and 5,6-epoxycholesterol [230] and formation of 5*α*-OOH [224]
Phospholipids	Main component of biological membranes	Plasma oxidizes phospholipids and affects lipid mobility [104, 332] Plasma induces apoptosis and flipping of phosphatidylserine from the inner to the outer layer of the cell membrane [140, 236, 238, 333–335] Plasma-treated cells present disrupted cell membranes [336–338]	Apoptotic cells presenting OxPLs in the cell membrane are eliminated by M2 macrophages [234]
Fatty acids	Form the hydrophobic hydrocarbon tails of phospholipids	Oxidation product NO_2_-FAs inhibit activation of NF*κ*B [188]	NO_2_-FAs stop the lipid oxidation propagation and protein nitration [240]. Peroxidation increases the rigidity of the cell membrane [339]
Lipid rafts	Modulate distribution of receptors and signalling molecules in the cell membrane [340] Important in oxidative stress-induced cell death [341]	In combination with hyperthermia, plasma activates the FA receptor (abundant in lipid rafts) and causes FA-induced apoptosis [342]	Activation and aggregation of death receptors such as FAs and TNFR1 located in lipid rafts and enhanced activation of kinases recruited at the raft site [341]. Ceramides produced from the oxidation of glycosphingolipids induce apoptosis via activation of the JNK pathway and regulation of Bax [343] and bind to cathepsin D to mediate TNF-induced cell death signalling [344]. In response to H_2_O_2_, JNK activates to induce the TRAF2/RIP-dependent pathway for oxidative cell death [341]. Lipid peroxidation affects the coupling of receptors with effector systems and decreases receptor density [339]

Catalytic enzymes			
NADPH oxidase (Nox)	Transmembrane enzyme that catalyzes the reduction of extracellular oxygen to O_2_^•−^	Inhibition with DPI attenuates the intracellular presence of ROS after plasma treatment, indicating a stimulation of endogenous ROS production with plasma [14]	Contributes to the elimination of malignant cells via HOCl and the NO/ONOO^−^ signalling pathways
Catalase	Membrane-bound enzyme that decomposes H_2_O_2_ into water and oxygen. When membrane-bound, it provides increased resistance to exogenous H_2_O_2_ and favors tumor progression	Plasma-generated ROS supposedly induce the formation of singlet oxygen that inactivates membrane-bound catalase to favor apoptosis [345]	In malignant cells, catalase interferes with HOCl signalling by decomposing H_2_O_2_ and interferes with NO/ONOO^−^ signalling through oxidation of NO and decomposition of ONOO^−^ to favor tumor progression
